# An Imbalance in Histone Modifiers Induces tRNA-Cys-GCA Overexpression and tRF-27 Accumulation by Attenuating Promoter H3K27me3 in Primary Trastuzumab-Resistant Breast Cancer

**DOI:** 10.3390/cancers16061118

**Published:** 2024-03-11

**Authors:** Ningjun Duan, Yijia Hua, Xueqi Yan, Yaozhou He, Tianyu Zeng, Jue Gong, Ziyi Fu, Wei Li, Yongmei Yin

**Affiliations:** Department of Oncology, First Affiliation Hospital of Nanjing Medical University, Nanjing 210029, China; henry-hua@outlook.com (Y.H.); yxqsdhr@163.com (X.Y.); heyaozhou111@outlook.com (Y.H.); drtianyu@163.com (T.Z.); gongjue@stu.njmu.edu.cn (J.G.); ziyi.fu@njmu.edu.cn (Z.F.); real.lw@163.com (W.L.)

**Keywords:** breast cancer, trastuzumab resistance, tRNA derived fragments, tRNA transcription, histone modification

## Abstract

**Simple Summary:**

tRF-27 has been identified as upregulated in both *naïve* trastuzumab-resistant HER2-positive breast cancer cells and patient samples, positioning it as a potential biomarker. However, the underlying mechanisms of this upregulation remain unclear. This study proposes that the abnormal transcription of specific tRNA-Cys-GCA isodecoders in *naïve* trastuzumab-resistant cells might contribute to the accumulation of tRF-27. Additionally, the reduction in H3K27me3 modifications at these tRNA genes, attributed to imbalances in histone modification enzymes, appears to influence tRNA transcription alterations. Building upon these findings, our research demonstrates that the application of a demethylase inhibitor may offer a potential strategy to overcome trastuzumab resistance.

**Abstract:**

tRNA-derived fragments (tRFs) play crucial roles in cancer progression. Among them, tRF-27 has been identified as a key factor in promoting *naïve* trastuzumab resistance in HER2-positive breast cancer. However, the origin of tRF-27 remains uncertain. In this study, we propose that the upregulated expression of specific cysteine tRNAs may lead to the increased accumulation of tRF-27 in trastuzumab-resistant JIMT1 cells. Mechanistically, the reduced inhibitory H3K27me3 modification at the promoter regions of tRF-27-related tRNA genes in JIMT1 cells, potentially resulting from decreased EZH2 and increased KDM6A activity, may be a critical factor stimulating the transcriptional activity of these tRNA genes. Our research offers fresh insights into the mechanisms underlying elevated tRF-27 levels in trastuzumab-resistant breast cancer cells and suggests potential strategies to mitigate trastuzumab resistance in clinical treatments.

## 1. Introduction

Breast cancer has now emerged as a leading threat to women’s health worldwide, with nearly 25% of all breast cancer cases falling under the HER2-positive type [1]. Trastuzumab-based anti-HER2 treatments have been widely adopted in the management of HER2-positive breast cancer, effectively extending the lifespan of patients [2,3]. Nevertheless, a significant challenge persists in the treatment of certain HER2-positive breast cancer cases that show poor or even no response to trastuzumab [4].

One prominent hallmark of HER2-positive breast cancer is the overexpression of HER2, which sustains the generation of signals promoting cell survival and proliferation through self-dimerization and the activation of downstream pathways, including PI3K-AKT-mTOR and RAS-RAF cascades [5]. Trastuzumab, specifically designed to target the extracellular domain of HER2, serves to disrupt HER2 dimerization and subsequently inhibits the generation of downstream signals [6]. Up to this point, various potential mechanisms have been explored, including, but not limited to, HER2 mutations and the activation of bypass signaling pathways, in the context of trastuzumab resistance in HER2-positive breast cancers [4]. These studies have shed light on the promising role of CDK4/6 and PI3K inhibitors, which can significantly enhance the treatment of trastuzumab-resistant HER2-positive breast cancers [5,7]. Nevertheless, the ongoing efforts to uncover additional potential mechanisms contributing to trastuzumab resistance continue.

Small RNAs, such as small-interfering RNA and microRNA, are non-coding RNA molecules with less than 200 nucleotides. They play crucial roles in mRNA degradation and translation inhibition, functioning alongside proteins, particularly the Argonaute protein, within the RNA-induced silencing complex (RISC) [8]. Recently, a distinct group of small non-coding RNAs known as tRNA-derived fragments (tRFs) has been discovered. These tRFs exhibit remarkable sequence consistency with tRNAs. The origin of tRFs is commonly attributed to the programmed cleavage of specific tRNAs by nucleases, such as Angiogenin, particularly under stress conditions [8,9]. This property underscores their potential as valuable biomarkers in the field of disease diagnosis. The functions of tRFs are diverse. They can bind to the 3’ untranslated regions (UTRs) of specific mRNAs, thereby influencing protein synthesis in conjunction with YBX protein [10], interact with long non-coding RNAs through sequence complementarity, interfering with their biological functions [11], and directly interact with proteins like G3BP, thus regulating their activities [12].

Abnormal changes in tRFs are closely linked to various diseases, especially tumors, playing roles in their initiation, promotion, progression, metastasis, and the development of drug resistance during tumor treatment [9,13,14]. This underscores the promising potential of tRFs in the field of tumor research. In the present day, the majority of tRF-associated studies primarily concentrate on their regulatory functions within biological processes. However, only a limited number of these studies are dedicated to exploring their origins. This leaves us with numerous unanswered questions.

Previously, we have suggested an unusually high accumulation of tRF-27 in primary trastuzumab-resistant breast cancer [15], which makes it a possible predictive biomarker. We have also demonstrated its potential role in the abnormal activation of the HER2 signal cascade through interaction with G3BP2 and activating MTORC1 [12]. So, in this study, our objective was to uncover the reasons behind the higher levels of tRF-27 in *naïve* trastuzumab-resistant JIMT1 cells compared to sensitive SKBR3 cells. We demonstrated that the elevated expression of specific tRNA types in JIMT1 cells contributes to the accumulation of tRF-27. Mechanistically, a reduction in the promoter H3K27me3 level appears to stimulate the transcription of these tRNA genes, which is influenced by decreased methyltransferase EZH2 and increased demethylase KDM6A activity. Furthermore, the application of KDM6A inhibitor GSK-J4 can reduce the expression of these tRNAs and downstream tRF-27, ultimately enhancing the sensitivity of JIMT1 cells to trastuzumab. Our findings not only suggest a potential mechanism leading to tRF-27 accumulation in trastuzumab-resistant HER2-positive breast cancer cells, but also provide a possible method to alleviate trastuzumab resistance in clinical treatment.

## 2. Materials and Methods

### 2.1. Cell Culture

Primary HER2-positive trastuzumab-sensitive breast cancer SKBR3 cells and trastuzumab-resistant breast cancer JIMT1 cells were obtained from American Type Culture Collection (ATCC) and cultured in modified Eagle’s medium (DMEM) (Gibco, Waltham, MA, USA, 11960044) supplemented with 10% fetal bovine serum (FBS) (Gibco, 16140071), 80 U/mL penicillin, and 0.08 mg/mL streptomycin (Gibco, 15140122) at 37 °C in a humidified environment with 5% CO_2_.

### 2.2. Construction of tRNA Expression Plasmids and Transfection

U6 cassette tRNA expression plasmids were constructed based on pcDNA3.1, in which the CMV promoter was replaced by the U6 promoter and oligo dT sequence. Sequences of tRNA genes were cloned from the genome DNA extracted from JIMT1 cells and inserted into the U6 cassette with BamHI/HindIII double digestion and ligation. tRNA expression plasmids were amplified in the *E. coli* JM109 strain and extracted with NucleoBond Xtra Midi EF kit (MACHEREY-NAGEL, Dueren, Germany, 740420.50).

Transient transfection was performed with Lipofectamine 3000 (Thermo, Waltham, MA, USA, L3000001) following the manufacturer’s protocol. DNA or siRNA together with P3000 were diluted in Opti-MEN and mixed with diluted Lipofectamine 3000, then added to cells. After 48 h incubation, treated cells could be harvested for the following experiments.

### 2.3. Cell Viability Assay

Cell viability was performed via CCK-8 assay. First, 5000 cells were seeded in a 96-well plate. After 24 h incubation at 37 °C in a humidified environment with 5% CO_2_, the old medium was replaced by a new drug-containing medium. After another 48 h culture, the new medium with CCK-8 reagent (Vazyme, Nanjing, China, A311) was applied to replace the old medium. After 1 h incubation, the absorbance of each well at 450 nm wavelength was measured with a multi-well plate reader.

### 2.4. Western Blot

Cells were washed with PBS after collection and lysed in SDS lysis solution supplied with protein inhibitor cocktail (Beyotime, Shanghai, China, P0013G) on ice for 5 min. Lysates were centrifuged at 12,000 rpm for 10 min at 4 °C. The supernatants were mixed with 5× loading buffer (Beyotime, P0015) and boiled at 95 °C for 15 min. Proteins were separated by SDS-PAGE with 15% percentage gels, transferred to PVDF membranes (Bio-Rad, Hercules, CA, USA, 1620177) and blocked in 5% skim milk in TBST (20 mM Tris, 150 mM NaCl, and 0.1% Tween-20) for 1 h. Membranes were incubated in diluted primary antibodies (H3K27me3 (CST, C36B11), EZH2 (Abcam, Cambridge, UK, ab307646), KDM6A (Abcam, ab300513), and Histone H3 (Abcam, ab1791)) at 4 °C overnight. After washing with TBST 3 times, membranes were incubated in diluted horseradish peroxidase (HRP)-conjugated anti-rabbit secondary antibody at room temperature for 1 h. After being washed with TBST 3 times, protein bands were visualized in a Western Blot imaging system with an ECL chemiluminescence kit (Vazyme, E422-01) and quantified using ImageJ (v1.54).

### 2.5. RNA Extraction and Reverse Transcription

Total RNA was extracted with TRIzol reagent (Thermo, 15596026) following the manufacture’s protocol. Then, 0.5 mL TRIzol reagent was added to each well of a 6-well plate to lyse the cells. After adding 250 μL isopropanol to the aqueous phase and undergoing a 10 min incubation, total RNA was harvested by 10 min of centrifuging at 12,000× *g* at 4 °C. The RNA pellet was washed with 75% ethanol and solved in RNase-free water, and the following cDNA synthesis was performed with HiScript III RT SuperMix (Vazyme, R323-01).

### 2.6. Chromatin Immunoprecipitation

Cells were firstly fixed with 10 mL culture medium containing 1% formaldehyde and 1.1 mL of 1.25 M glycine. Chromatin extract was performed with a Chromatin Extraction Kit (Abcam, ab117152) following the manufacture’s protocol. In general, fixed cells were resuspend with lysis buffer on ice for 10 min, vortexed for 10 s and centrifuged at 5000× *g* rpm for 5 min. After removing the supernatant, extraction buffer was added to resuspend the chromatin pellet and it was incubated on ice for 10 min, followed by 10 min sonication, 2 s pulse/2 s pause, and centrifuging at 12,000× *g* rpm at 4 °C for 10 min. Chromatin immunoprecipitation was performed with one-step ChIP Kit (Abcam, ab117138). Briefly, sheared chromatin, together with a chip buffer and antibody (H3K27me3, EZH2, and KDM6A), was added in a strip well and incubated at room temperature for 120 min on an orbital shaker (100 rpm), and then the solution in each well was removed and washed with wash buffer 4 times, and the DNA released by adding DNA release buffer supplied with proteinase K and incubating at 65 °C for 20 min.

### 2.7. RNA-Seq Data Acquisition and Analysis

Transcriptome data were acquired from GSE236750, and tRFs expression data were acquired from GSE107473. Differential expression was analyzed using DESeq2. Significantly changed genes were defined as those with Padj ≤ 0.05 and |log2foldchange| ≥ 1.

### 2.8. Animal Study

JIMT1 cells were injected into the lower flanks of 6-week-old BALB/c nude mice. When the tumor volume reached approximately 40 mm^3^, mice were treated with trastuzumab (10 mg/kg) or PBS at the 1st and 4th days of each week and GSK-J4 (100 mg/kg) or DMSO from the 1st to 5th days of each week for 4 weeks via intraperitoneal injection. Tumor volume was calculated as 0.5 × width^2^ × length.

### 2.9. Statistics

Statistical analyses were performed with GraphPad Prism (v10). For bar plots, means ± standard deviation were indicated above. Student’s test was applied for comparison between two groups and ANOVA was applied for comparison between groups in the animal study. *p* < 0.05 was considered as the limit of significance and marked as * *p* < 0.05, ** *p* < 0.01, *** *p* < 0.001.

## 3. Results

### 3.1. Overexpression of tRNA-Cys-GCAs Causing tRF-27 Accumulation

**Figure 1:**
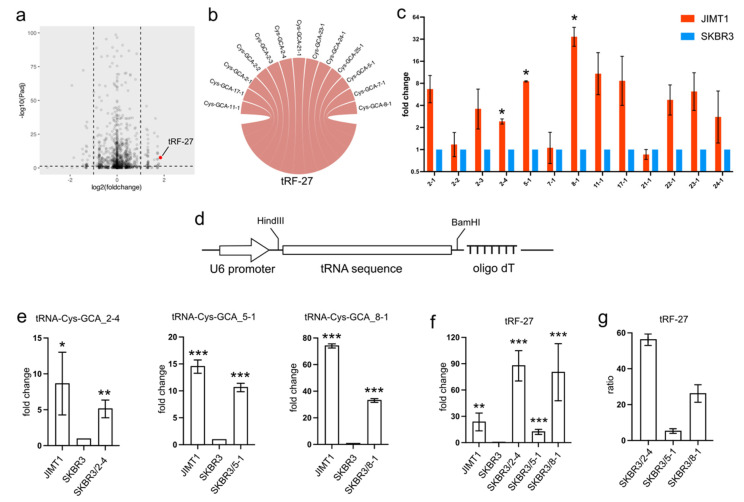
The overexpression of tRNA-Cys-GCAs stimulates tRF-27 accumulation. (**a**) The expression of tRF-27 in trastuzumab-resistant JIMT1 cells and trastuzumab-sensitive SKBR3 cells. (**b**) Isodecoders of tRNA-Cys-GCA have consistent sequence with tRF-27. (**c**) The expression of tRF-27-related tRNA-Cys-GCA in JIMT1 and SKBR3 cells. (**d**) The structure of the tRNA expression cassette, including the U6 promoter and oligo dT RNA pol III terminator. (**e**) The overexpression of tRNA-Cys-GCA_2-4, tRNA-Cys-GCA_5-1, and tRNA-Cys-GCA_8-1 in JIMT1 cells. (**f**) tRF-27 level after overexpression of three tRNA-Cys-GCAs. (**g**) The ratio between overexpressed tRNA-Cys-GCA and increased tRF-27. Three replicates were applied in each experiment. *t*-test was applied for statistical analysis. Data are shown as Mean ± SD. * *p* < 0.05, ** *p* < 0.01, *** *p* < 0.001.

In trastuzumab-resistant JIMT1 cells, tRF-27 exhibited an upregulation compared to trastuzumab-sensitive SKBR3 cells (Figure 1a). To trace the origin of tRF-27, we conducted sequence mapping, revealing its identity with several isodecoders of cysteine-associated tRNA. Specifically, the tRF-27 sequence showed similarity to isodecoders such as tRNA-Cys-GCA_2-1, tRNA-Cys-GCA_2-2, tRNA-Cys-GCA_2-3, tRNA-Cys-GCA_2-4, tRNA-Cys-GCA_5-1, tRNA-Cys-GCA_7-1, tRNA-Cys-GCA_8-1, tRNA-Cys-GCA_11-1, tRNA-Cys-GCA_21-1, tRNA-Cys-GCA_22-1, tRNA-Cys-GCA_23-1, and tRNA-Cys-GCA_24-1 (Figure 1b).

Given that tRFs are typically regarded as cleavage products of tRNAs, any abnormal expression of tRNAs could potentially impact the levels of tRFs. Our investigation involved assessing the expression of tRF-27-associated tRNA-Cys-GCAs in both JIMT1 and SKBR3 cells. Notably, we observed an overall upregulation of these tRNAs in trastuzumab-resistant cells, with particular prominence seen in tRNA-Cys-GCA_2-4, tRNA-Cys-GCA_5-1, and tRNA-Cys-GCA_8-1 (Figure 1c). To explore whether the increased expression of these three tRNAs could stimulate tRF-27 production, we conducted separate overexpression experiments for each tRNA using a small RNA expression plasmid featuring a U6 promoter and an oligo dT terminal signal in SKBR3 cells (Figure 1d). As anticipated, the final levels of the three tRNAs increased in the transfected SKBR3 cells (Figure 1e), consequently leading to an elevation in the downstream tRF-27 (Figure 1f). Upon comparing the altered levels of tRF-27 and the relevant tRNAs, we noted that tRNA-Cys-GCA_2-4 exhibited a stronger capacity to generate tRF-27 compared to tRNA-Cys-GCA_8-1 and tRNA-Cys-GCA_5-1 (Figure 1g). These findings underscore the potential regulatory role of specific tRNAs in influencing tRF-27 production in the context of trastuzumab resistance.

### 3.2. Reduced Promoter H3K27me3 Stimulates tRNA Transcription

The transcriptional activity of tRNA genes is subject to regulation through various mechanisms, encompassing protein factors that modulate RNA pol III activity and epigenetic modifications at the promoter regions of tRNA genes.

**Figure 2:**
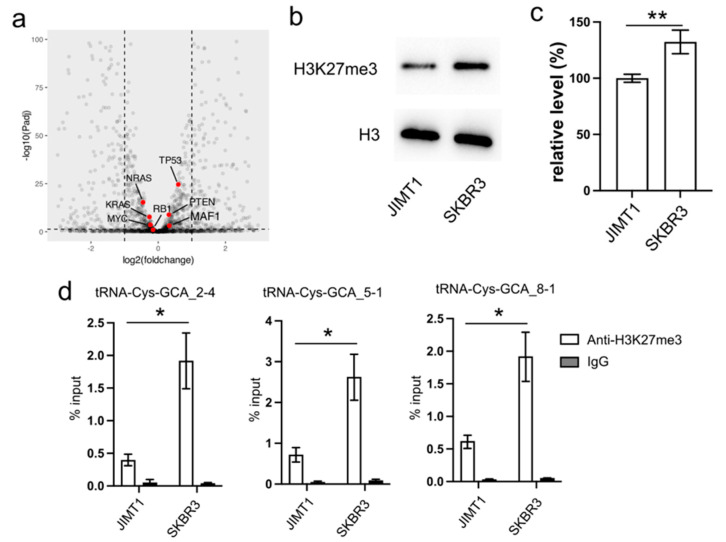
Reduced cellular H3K27me3 stimulates tRNA-Cys-GCA transcription. (**a**) The transcription states of several RNA pol III regulators in JIMT1 and SKBR3 cells. (**b**,**c**) The overall level of H3K27me3 in JIMT1 and SKBR3 cells. (**d**) The enrichment of H3K27me3 at the promoters of three tRNA-Cys-GCAs in JIMT1 and SKBR3 cells. Three replicates were applied in each experiment. T-test was applied for statistical analysis. Data are shown as Mean ± SD. * *p* < 0.05, ** *p* < 0.01. The uncropped blots are shown in Figure S3.

In our investigation, we initially examined the expression of several proteins known to regulate RNA pol III activity, such as MAF1, RB1, TP53, and RAS. However, their expression levels exhibited limited differences between trastuzumab-sensitive and resistant cells (Figure 2a).

Turning our attention to the impact of epigenetic modifications on gene transcription, we explored the inhibitory role of tri-methylated H3K27 at the promoter region of tRNA genes. Our Western blot results revealed an overall lower density of H3K27me3 in JIMT1 cells compared to SKBR3 cells (Figure 2b,c, Supplementary Figure S3). Furthermore, through ChIP-qPCR analysis, we identified a higher enrichment of H3K27me3 modification at the promoter regions of tRNA-Cys-GCA_2-4, tRNA-Cys-GCA_5-1, and tRNA-Cys-GCA_8-1 in SKBR3 cells compared to JIMT1 cells (Figure 2d). This discrepancy suggests a potential mechanism wherein increased H3K27me3 modification acts to repress the transcription of these specific tRNA genes, shedding light on a regulatory pathway influencing trastuzumab resistance.

### 3.3. Downregulated EZH2 Promotes tRNA-Cys-GCA Overexpression

EZH2 serves as the primary methyltransferase involved in the tri-methylation of H3K27, and alterations in its activity can influence the H3K27me3 levels in gene promoters, thereby regulating gene transcription.

**Figure 3:**
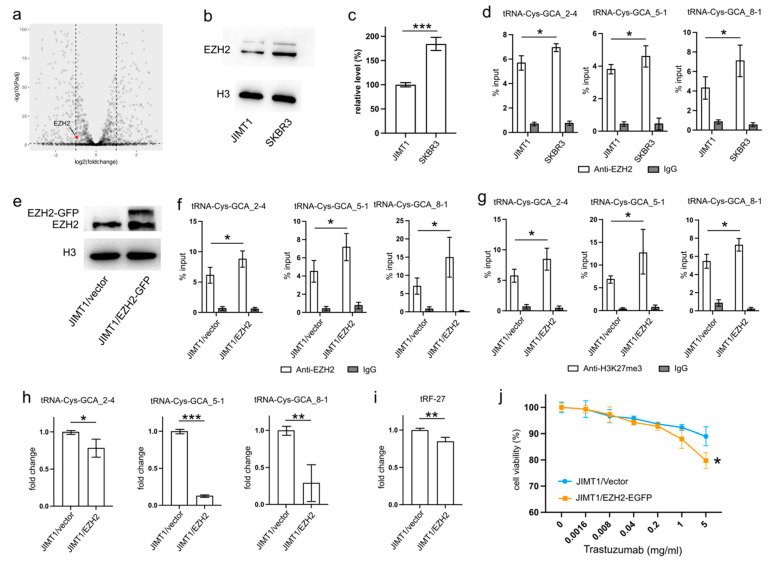
Downregulated EZH2 increases tRNA-Cys-GCA transcription and tRF-27 accumulation. (**a**) The transcription states of EZH2 in JIMT1 and SKBR3 cells. (**b**,**c**) The protein level of EZH2 in JIMT1 and SKBR3 cell nucleus. (**d**) The enrichment of EZH2 at the promoters of three tRNA-Cys-GCAs in JIMT1 and SKBR3 cells. (**e**) The result of EZH2 overexpression in JIMT1 cell nucleus. (**f**) The enrichment of EZH2 at the promoters of three tRNA-Cys-GCAs in JIMT1 and EZH2-overexpressed JIMT1 cells. (**g**) The enrichment of H3K27me3 at the promoters of three tRNA-Cys-GCAs in JIMT1 and EZH2-overexpressed JIMT1 cells. (**h**) The transcription of tRF-27-related tRNA-Cys-GCAs in JIMT1 and EZH2-overexpressed JIMT1 cells. (**i**) The level of tRF-27 in JIMT1 and EZH2-overexpressed JIMT1 cells. (**j**) The viabilities of JIMT1 and EZH2-overexpressed JIMT1 cells treated with different concentrations of trastuzumab. Three replicates were applied in each experiment. T-test and ANOVA were applied for statistical analysis. Data are shown as mean ± SD. * *p* < 0.05, ** *p* < 0.01, *** *p* < 0.001. The uncropped blots are shown in Figure S3.

An analysis of transcriptomic data revealed a higher level of EZH2 in SKBR3 cells compared to JIMT1 cells (Figure 3a). This trend was further substantiated by Western blot results, confirming a similar pattern of nuclear EZH2 levels (Figure 3b,c, Supplementary Figure S3). To investigate whether the lower nuclear EZH2 levels in JIMT1 cells could impact the tri-methylation of H3K27 in the promoters of tRF-27-related tRNA genes, we performed ChIP-qPCR with an anti-EZH2 antibody. The results confirmed a higher enrichment of EZH2 at these tRNA promoters in SKBR3 cells, consistent with the distribution of H3K27me3 signals (Figure 3d).

To validate the regulatory role of EZH2 in tRNA expression, we transfected JIMT1 cells with an EZH2 overexpression plasmid. The nuclear EZH2 levels in transfected JIMT1 cells increased compared to non-transfected cells (Figure 3e, Supplementary Figure S3). Subsequently, we observed an upregulated enrichment of EZH2 at the promoter regions of the three tRF-27-related tRNA genes (Figure 3f), along with a higher enrichment of H3K27me3 at these promoter regions in EZH2-overexpressed JIMT1 cells (Figure 3g). Consequently, the transcriptional activities of tRNA-Cys-GCA_2-4, tRNA-Cys-GCA_5-1, and tRNA-Cys-GCA_8-1 were inhibited in EZH2-overexpressed JIMT1 cells, with tRNA-Cys-GCA_5-1 exhibiting the greatest decrease in expression (Figure 3h). Simultaneously, the cellular level of tRF-27, as well as trastuzumab sensitivity, were reduced compared to the original JIMT1 cells (Figure 3i,j). Furthermore, we observed a decrease in promoter H3K27me3 levels and increased transcriptional activities of three tRNA genes in SKBR3 cells with reduced EZH2 (Supplementary Figures S1a–d and S3). This was accompanied by heightened levels of tRF-27 and an associated increase in trastuzumab resistance (Supplementary Figure S1e,f).

These findings underscore the regulatory impact of EZH2 on tRNA expression and its downstream consequences on tRF-27 levels in the context of trastuzumab resistance.

### 3.4. Upregulated KDM6A Stimulates tRNA-Cys-GCA Overexpression

The genomic H3K27me3 level is intricately regulated by the activities of both the methyltransferase EZH2 and the demethylases KDM6A and KDM6B. Thus, altered activities of KDM6A and KDM6B can potentially contribute to changes in the promoter H3K27me3 levels of tRNA genes.

**Figure 4:**
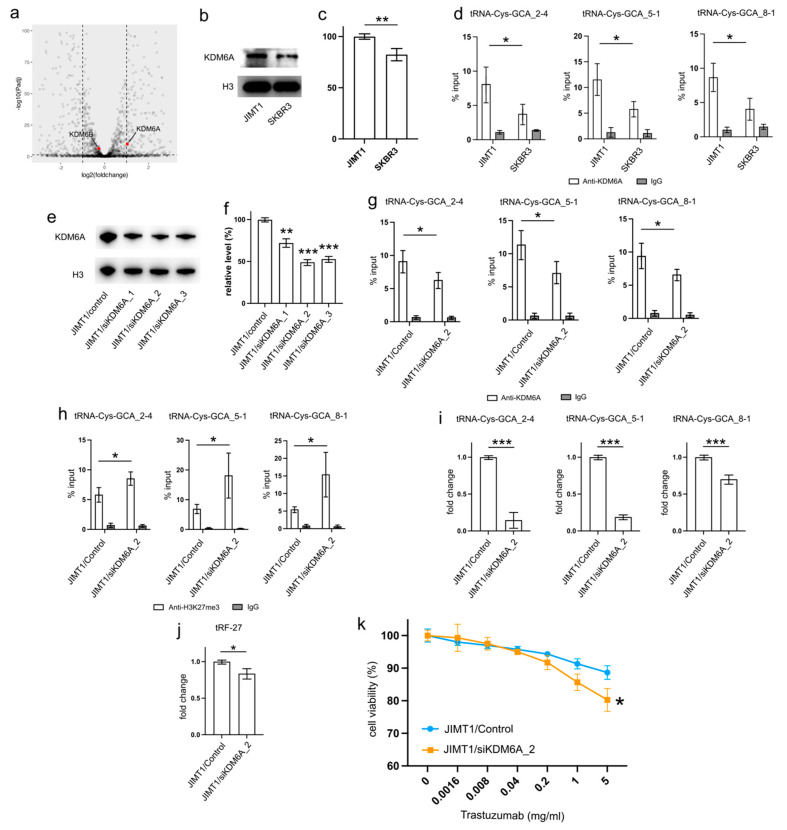
Upregulated KDM6A increased tRNA-Cys-GCA transcription and tRF-27 accumulation. (**a**) The transcription states of KDM6A and KDM6B in JIMT1 and SKBR3 cells. (**b**,**c**) The protein level of KDM6A in JIMT1 and SKBR3 cell nucleus. (**d**) The enrichment of KDM6A at the promoters of three tRNA-Cys-GCAs in JIMT1 and SKBR3 cells. (**e**,**f**) KDM6A knockdown in JIMT1 cell nucleus. (**g**) The enrichment of KDM6A at the promoters of three tRNA-Cys-GCAs in JIMT1 and KDM6A-knockdown JIMT1 cells. (**h**) The enrichment of H3K27me3 at the promoters of three tRNA-Cys-GCAs in JIMT1 and KDM6A-knockdown JIMT1 cells. (**i**) The transcription of tRF-27-related tRNA-Cys-GCAs in JIMT1 and KDM6A-knockdown JIMT1 cells. (**j**) The level of tRF-27 in JIMT1 and KDM6A-knockdown JIMT1 cells. (**k**) The viabilities of JIMT1 and KDM6A-reduced JIMT1 cells treated with different concentrations of trastuzumab. Three replicates were applied in each experiment. T-test and ANOVA were applied for statistical analysis. Data are shown as Mean ± SD. * *p* < 0.05, ** *p* < 0.01, *** *p* < 0.001. The uncropped blots are shown in Figure S3.

Examining the transcriptomic data revealed a stable expression of KDM6B in both JIMT1 and SKBR3 cells, while KDM6A exhibited a higher expression in JIMT1 cells compared to SKBR3 cells (Figure 4a). This trend was further supported by Western blot results, which showed a higher nuclear KDM6A level in JIMT1 cells as opposed to SKBR3 cells (Figure 4b,c, Supplementary Figure S3). Investigating the KDM6A levels in the promoters of tRF-27-relevant tRNA genes through ChIP-qPCR, we observed a more pronounced enrichment of KDM6A in JIMT1 cells compared to SKBR3 cells. This observation suggests the possibility that increased KDM6A levels could contribute to the reduction of H3K27me3 at these tRNA gene promoters (Figure 4d).

To corroborate the impact of high KDM6A levels on the transcriptional regulation of tRNA genes, we employed a set of anti-KDM6A siRNAs to knock down its expression in JIMT1 cells. The successful reduction in nuclear KDM6A levels was observed after 48 h of transfection (Figure 4e,f, Supplementary Figure S3). A subsequent analysis of the most reduced group indicated a downregulation of KDM6A enrichment at the promoter regions of three tRNA genes (Figure 4g). Moreover, an increase in tri-methylated H3K27 enrichment at the promoter regions of these three tRNA genes was detected (Figure 4h). Consequently, a reduction in the expression of the three tRNA genes was observed, with tRNA-Cys-GCA_2-4 and tRNA-Cys-GCA_5-1 showing a stronger decrease than tRNA-Cys-GCA_8-1 (Figure 4i). Simultaneously, the cellular tRF-27 level and trastuzumab resistance was reduced in KDM6A knockdown JIMT cells (Figure 4j,k). Concurrently, we noted a reduction in the promoter H3K27me3 levels and enhanced transcriptional activities of three tRNA genes in SKBR3 cells with overexpressed KDM6A (Supplementary Figures S2a–c and S3). This correlated with an escalation in both tRF-27 levels and resistance to trastuzumab (Supplementary Figure S2d,e).

These results emphasize the role of KDM6A in influencing tRNA gene expression and subsequently impacting tRF-27 levels in the context of trastuzumab resistance.

### 3.5. GSK-J4 Reduces tRF-27 Production and Relieves Trastuzumab Resistance

**Figure 5:**
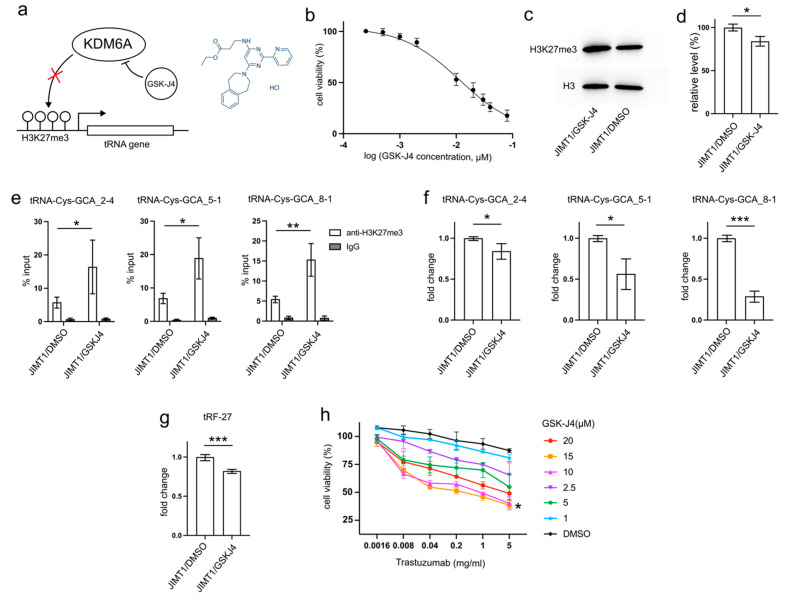
GSK-J4 reduces tRNA-Cys-GCA transcription and tRF-27 accumulation. (**a**) The principle that GSK-J4 inhibits the activity of KDM6A and diminishes H3K27me3. (**b**) The viabilities of JIMT1 cells treated with different concentrations of GSK-J4. (**c**,**d**) The overall H3K27me3 level in JIMT1 and GSK-J4-treated JIMT1 cells. (**e**) The enrichment of KDM6A at the promoters of three tRNA-Cys-GCAs in JIMT1 and GSK-J4-treated JIMT1 cells. (**f**) The transcription of tRF-27-related tRNA-Cys-GCAs in JIMT1 and GSK-J4-treated JIMT1 cells. (**g**) The level of tRF-27 in JIMT1 and GSK-J4-treated JIMT1 cells. (**h**) The viabilities of JIMT1 cells treated with different concentrations of GSK-J4 and trastuzumab. Three replicates were applied in each experiment. T-test and ANOVA were applied for statistical analysis. Data are shown as Mean ± SD. * *p* < 0.05, ** *p* < 0.01, *** *p* < 0.001. The uncropped blots are shown in Figure S3.

GSK-J4, developed as a specific inhibitor of KDM6A/B to enhance H3K27 methylation, has demonstrated anti-cancer abilities by reducing tumor cell proliferation and inducing apoptosis in various studies [16,17,18,19]. This inhibitor may also play a role in decreasing tRNA transcription (Figure 5a).

After 48 h incubation with 10μM GSK-J4, a concentration slightly above its IC_50_ (11.93 μM) on JIMT1 cells (Figure 5b), treated JIMT1 cells displayed an increment in global H3K27me3 levels compared to untreated JIMT1 cells (Figure 5c,d, Supplementary Figure S3). Additionally, noticeable increases in H3K27me3 signals were observed at the promoter regions of all three tRF-27-associated tRNA genes (Figure 5e). As a result, the expressions of tRNA-Cys-GCA_2-4, tRNA-Cys-GCA_5-1, and tRNA-Cys-GCA_8-1 all experienced reductions (Figure 5f), while the cellular tRF-27 level also demonstrated a decrease after GSK-J4 treatment (Figure 5g).

Furthermore, we explored whether GSK-J4 could impact the trastuzumab tolerance of JIMT1 cells. We conducted viability assessments of JIMT1 cells under varying concentrations of GSK-J4 and trastuzumab (Figure 5h). Notably, both 10 μM and 15 μM concentrations of GSK-J4 demonstrated comparable effects in enhancing trastuzumab sensitivity, outperforming other concentrations.

These findings suggest a potential therapeutic strategy involving GSK-J4 to enhance trastuzumab effectiveness in treating HER2-positive breast cancer, possibly by influencing H3K27me3 levels and tRNA-related pathways.

### 3.6. GSK-J4 Inhibits Tumor Growth in a Xenograft Mouse Model

In addition to cell experiments, GSK-J4 has demonstrated efficacy in reducing tumor progression, both as a standalone treatment and in combination with other anti-cancer agents, in various animal studies [20,21,22].

**Figure 6:**
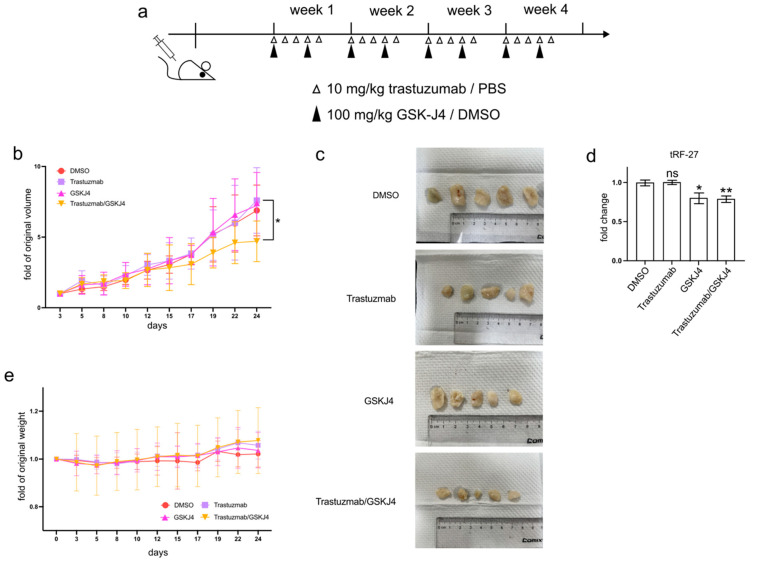
GSK-J4 reduces tumor growth on mice model. (**a**) The treatment strategies of mice in the animal experiment. (**b**) The tumor volume changes of four mouse groups during animal experiments (N = 5, 5, 5, 5). (**c**) The final tumor shapes of four mouse groups (N = 5, 5, 5, 5). (**d**) The relative tumor tRF-27 levels of four mouse groups (N = 5, 5, 5, 5). (**e**) The body weight changes of four mouse groups during animal experiments (N = 5, 5, 5, 5). *t*-test and ANOVA were applied for statistical analysis. Data are shown as Mean ± SD. ns *p* >= 0.05, * *p* < 0.05, ** *p* < 0.01.

To investigate whether GSK-J4 can mitigate trastuzumab resistance in HER2-positive breast cancer cells in vivo, we established xenograft mouse models by transplanting JIMT1 cells into the lower flanks of BALB/c nude mice and treating them with trastuzumab and/or GSK-J4 (Figure 6a). Our findings indicated that single treatment groups with either trastuzumab or GSK-J4 effectively slowed down the growth rate of tumors compared to the double-negative treatment group. However, the co-treatment group with trastuzumab and GSK-J4 began to show a decreased growth rate from the 17th day and ultimately demonstrated a reduction in tumor volume (Figure 6b,c). Correspondingly, the levels of tRF-27 were lower in the GSK-J4 treated groups than in the untreated groups (Figure 6d). Moreover, despite some fluctuations, the body weight of the co-treatment group remained stable, with almost no differences compared to the negative control group (Figure 6e).

These results suggest the potential of GSK-J4 in reducing trastuzumab resistance in HER2-positive breast cancer in an in vivo setting, highlighting its promising role as a therapeutic agent.

## 4. Discussion

tRNA-derived fragments (tRFs) are generated through the degradation of tRNAs, and disruptions in the tRNA cleavage processes and tRNA abundance are potential factors contributing to the abnormal accumulation of specific tRF types. Numerous studies have emphasized that the abnormal expression of the tRNA cleavage enzyme angiogenin [23,24], along with modifications in tRNAs that play a role in maintaining their stability by preventing nuclease cleavage, can lead to diverse patterns of tRF accumulation, particularly under stress conditions.

Although there has been a considerable amount of research on the influence of tRNA cleavage enzyme expression and modifications on tRF accumulation, fewer studies have delved into the relationship between the expression levels of tRNAs and the abundance of tRFs. Torres et al. conducted a noteworthy study that demonstrated that changes in tRNA gene expression can indeed lead to altered tRF abundances [25]. This research serves as a crucial contribution in systematically unraveling the connection between the expression levels of tRNAs and the presence of tRFs.

RNA polymerase III (RNA pol III) plays a crucial role in transcribing various small RNA genes, including tRNA genes. Similar to RNA polymerase II, the activity of RNA pol III undergoes direct regulation through signaling pathways and protein factors. Key regulatory players in this context encompass PI3K/AKT, mTOR, TP53, RAS/ERK, MYC, and MAF1, all of which exert control over RNA pol III activity and downstream tRNA gene expression [26,27]. Additionally, the indirect regulation of RNA pol III activity can occur through epigenetic modifications on genomic DNA and histones near specific RNA pol III-targeting genes [28].

The alterations in both whole-genomic and site-specific histone modifications are widely observed in each stage of cancer development. Throughout this process, epigenetic reprogramming, particularly the modification of H3K27me3, can regulate the expression of key elements associated with cancer cell survival, proliferation, and migration. The activity of the PI3K/AKT signaling cascade is regulated by PTEN, whose expression in glioblastoma can be inhibited by the upregulated promoter H3K27me3. This inhibition leads to the activation of the downstream AKT/mTOR pathway, subsequently promoting epithelial–mesenchymal transition (EMT) [29]. Additionally, the reduced expression of the WNT/β-catenin pathway antagonist, DKK1, is attributed to increased promoter H3K27me3. This alteration results in the overactivation of the WNT/β-catenin pathway, enhancing the pluripotency of cancer cells [30]. Notably, decreased H3K27me3 levels have been observed at the promoters of HES1 and HES5 genes, leading to the unusual activation of the NOTCH pathway and the subsequent stimulation of EMT [31]. Proteins that do not belong to the major signaling pathways still play vital roles in cancer cell survival, proliferation, and metastasis. Examples include RAD51, RUNX3, FOXC1, and CDH1, whose expression can also be regulated by H3K27me3 [32,33,34].

Furthermore, H3K27me3 plays a role in the transcriptional regulation of cancer-associated non-coding RNAs. Genomic-wide studies have indicated that numerous non-coding RNAs undergo changes in promoter H3K27me3 consistently with alterations in their expression [35]. The downregulation of H3K27me3 at the promoters of non-coding RNAs such as ANCR and DANCR can stimulate their expression, leading to invasion, metastasis, and poor prognosis [36,37]. While most studies have focused on the effects of H3K27me3 on non-coding RNAs transcribed by RNA pol II, our work extends this knowledge to the expression of small non-coding RNAs like certain types of tRNAs transcribed by RNA pol III. These small non-coding RNAs can also be regulated by alterations in H3K27me3, particularly in diseases such as breast cancer.

Several mechanisms have been proposed to explain the development of drug resistance during anti-cancer treatment, including but not limited to cell death inhibition, drug inactivation, and drug efflux [38]. Histone modification is a crucial factor in regulating the expression of key genes within these processes, influencing the drug resistance of cancer cells. The Cytochrome P450 system (CYP) comprises crucial drug metabolism enzymes, and the expression of its members, such as CYP3A16, can be suppressed by increased H3K27me3 [39]. Similarly, another drug metabolism enzyme, DKHS4, can have its expression silenced by H3K27me3 [40]. Another mechanism for effectively reducing drug concentration within cells involves the upregulation of exporters. The expression of the multidrug transporter SLC47A1, which is correlated with chemoresistance in cancer cells, is under the control of H3K27me3 modification [41].

**Figure 7:**
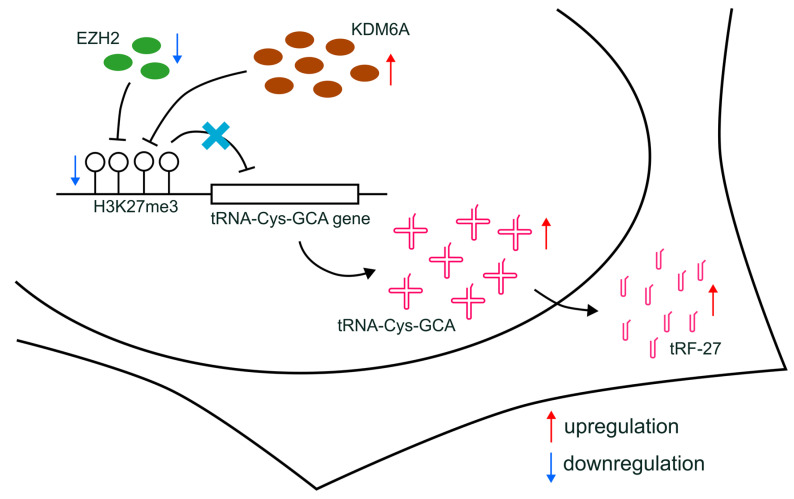
Schematic diagram of the proposed mechanisms. The downregulation of EZH2, combined with increased KDM6A expression, leads to the reduction in H3K27me3 levels at the promoters of tRNA-Cys-GCA genes and the upregulated transcription of tRNA-Cys-GCA, resulting in subsequently increased cellular levels of tRF-27.

Furthermore, altered H3K27me3 can influence drug resistance through indirect effects, such as modifying the expression of non-coding RNAs [42]. For instance, circRNA CDR1-AS, which regulates miR-7 function and affects tamoxifen resistance, has its transcription controlled by H3K27me3 accumulation [43]. Additionally, the expression of the long non-coding RNA (lncRNA) EPB41L4A-AS2, related to docetaxel sensitivity, can be regulated by H3K27me3 enrichment [44]. In our study, we proposed that the production of tRFs can be affected by the transcriptional regulation of relevant tRNAs, which, in turn, can be further regulated by alterations in H3K27me3 enrichment (Figure 7). Our findings shed light on a novel understanding of the indirect relationship between histone modification and drug resistance in breast cancer, providing a potential avenue for future research.

## 5. Conclusions

In our study, we propose that the decreased H3K27me3 level at the promoter regions of tRNA-Cys-GCA, potentially induced by the downregulation of EZH2 and the concurrent upregulation of KDM6A, could represent a plausible mechanism for enhancing the transcription of specific tRNA-Cys-GCA. This, in turn, leads to the accumulation of tRF-27 and ultimately contributes to primary trastuzumab resistance in HER2-positive breast cancer. 

## Data Availability

Data are contained within the article and Supplementary Materials.

## Supplementary Materials

The following supporting information can be downloaded at: https://www.mdpi.com/article/10.3390/cancers16061118/s1, Figure S1: EZH2 knockdown leads to tRF-27 accumulation; Figure S2: KDM6A overexpression leads to tRF-27 accumulation; Figure S3: Uncropped original western blot figures.
