# Supplementary material for: An Imbalance in Histone Modifiers Induces tRNA-Cys-GCA Overexpression and tRF-27 Accumulation by Attenuating Promoter H3K27me3 in Primary Trastuzumab-Resistant Breast Cancer
Source: Cancers (Basel). 2024 Mar 11;16(6):1118. doi: 10.3390/cancers16061118 (PMC10968641; doi:10.3390/cancers16061118)
Supplement: Supplementary file 1 [file cancers-16-01118-s001.zip › cancers-2805122-supplementary Figure S1.pdf]

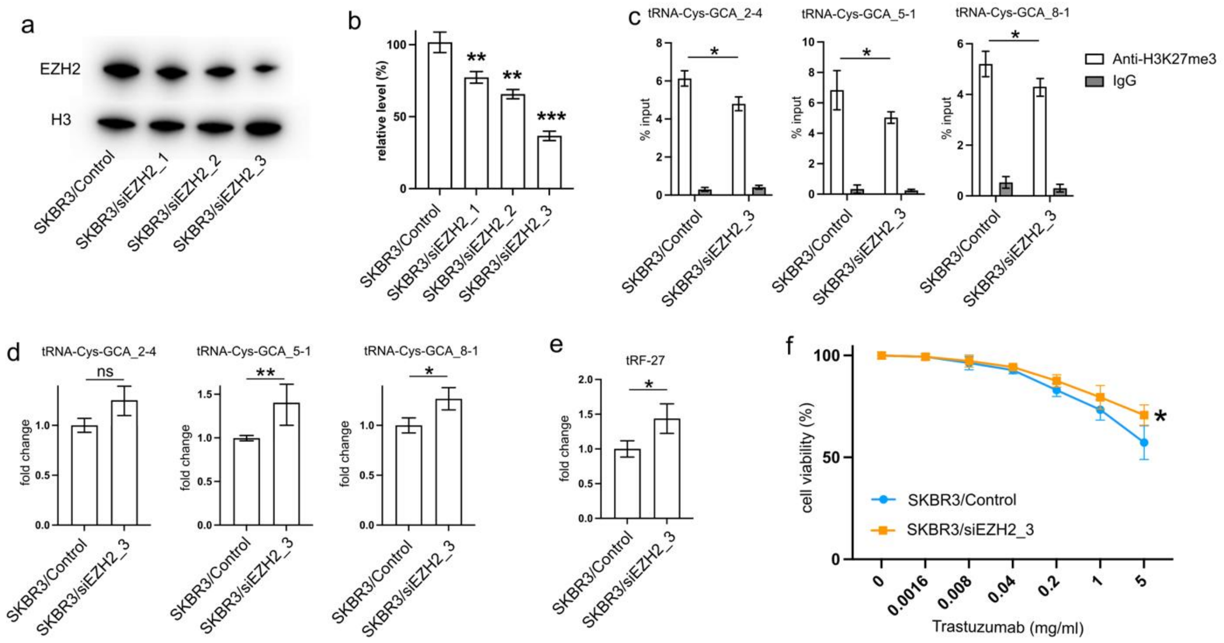

Supplementary Figure S1

a-b, The result of EZH2 knockdown in SKBR3 cell nucleus. **c,** The enrichment of H3K27me3 at the promoters of three tRNA-Cys-GCAs in SKBR3 and EZH2 knockdown SKBR3 cells. **d,** The transcription of tRF-27-related tRNA-Cys-GCAs in SKBR3 and EZH2 knockdown SKBR3 cells. **e,** The level of tRF-27 in SKBR3 and EZH2 knockdown SKBR3 cells. **f,** The viabilities of JIMT1 and EZH2 knockdown JIMT1 cells treated with different trastuzumab concentrations. Three replicates were applied in each experiment. T-test and ANOVA were applied for statistical analysis. Data are shown as Mean $\pm$ SD. $*P < 0.05$, $**P < 0.01$, $***P < 0.001$.
