# Supplementary figures and images for: An Imbalance in Histone Modifiers Induces tRNA-Cys-GCA Overexpression and tRF-27 Accumulation by Attenuating Promoter H3K27me3 in Primary Trastuzumab-Resistant Breast Cancer
Source: Cancers (Basel). 2024 Mar 11;16(6):1118. doi: 10.3390/cancers16061118 (PMC10968641; doi:10.3390/cancers16061118)

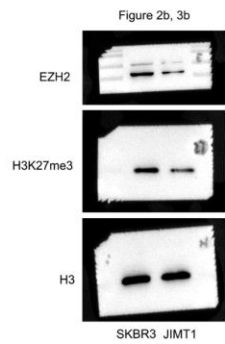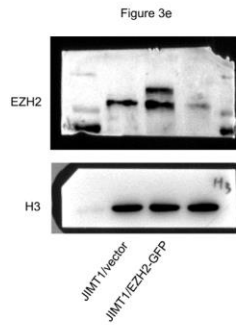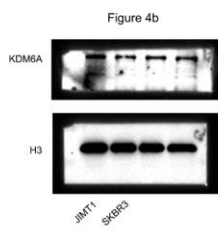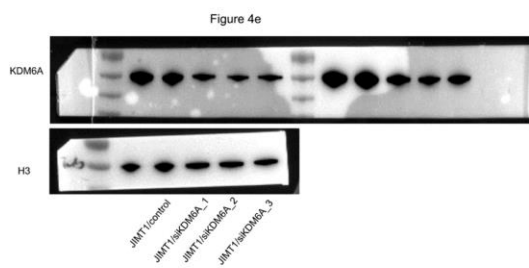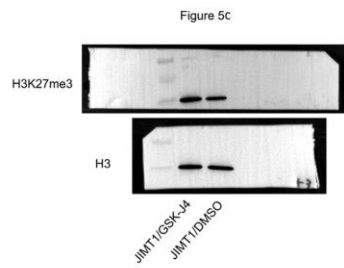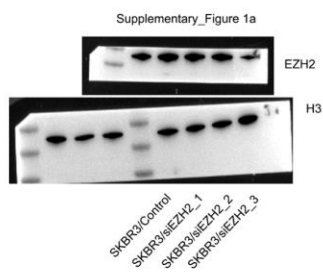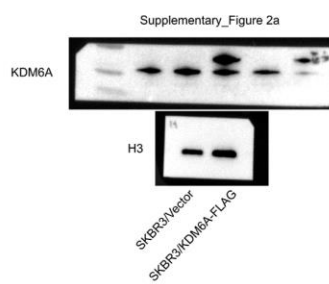

Supplement: Supplementary file 1 [file cancers-16-01118-s001.zip › cancers-2805122-supplementary Figure S3.pdf]
